# Utilization of nanosensors and nanomaterials to revolutionize the food industry: boosting product safety, quality, and sustainability

**DOI:** 10.1039/d6ra04714a

**Published:** 2026-07-09

**Authors:** Samah M. El-Sayed, Ahmed M. Youssef

**Affiliations:** a Dairy Science Department, National Research Centre 33 El Bohouth St. (former El Tahrir St.) P.O. 12622, Dokki Giza Egypt samah_mosbah80@yahoo.com; b Packaging Materials Department, National Research Centre 33 El Bohouth St. (former El Tahrir st.), P.O. 12622, Dokki Giza Egypt amyoussef27@yahoo.com Youssef@bnu.edu.eg; c Center for Converging Sciences and Emerging Technology (CoSET), Benha National University (BNU) Al Obour 13518 Egypt

## Abstract

Nanotechnology is revolutionizing the food industry by improving safety, quality, and sustainability through the use of nanosensors and nanomaterials. Several nanosensors are employed, including biosensors for rapid pathogen detection, nanocomposite indicators for food freshness, and quantum dot-based sensors for heavy metal and pesticide detection. Other types include cantilever-based sensors, carbon nanotube-based electrochemical sensors, nanowires, nano-electromechanical systems (NEMS), and luminous nanoparticle labels for targeted detection. These nanosensors outperform standard processes in terms of sensitivity, speed, and selectivity, allowing for real-time food quality monitoring, contamination detection, and spoiling indication. They also provide temperature monitoring, microbiological detection, and color change indicators in smart packaging. Some of the key benefits include improved quality through freshness monitoring, reduced food waste through accurate shelf-life indications, and increased food safety through early pathogen and toxin detection, and support for sustainability through improved supply chain management and safer packaging solutions. However, there are significant barriers to the application of nanotechnology in food and its integration into existing food industry operations.

## Abstract 2

Nanotechnology is revolutionizing the food industry by improving safety, quality, and sustainability through the use of nanosensors and nanomaterials.

## Graphical abstract

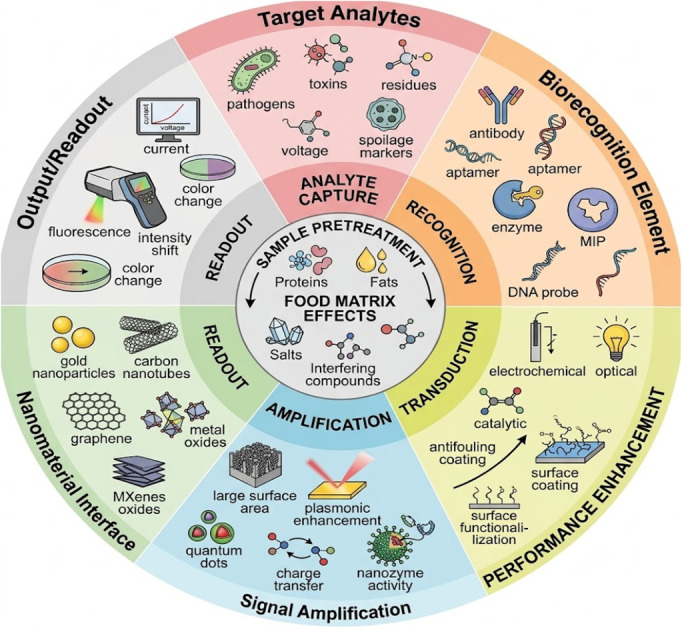

## 1 1-Introduction

Nanotechnology has transformed the food industry by enabling innovations in processing, packaging, and quality control, with applications that improve food safety, sustainability, and product quality.^1^ It can enhance nutrition and sensory properties through approaches such as nanoemulsions for better nutrient bioavailability and nanocarriers for protecting and delivering vitamins, minerals, and flavor compounds in a more stable and controlled way.^2–6^ In food packaging, nanotechnology supports active systems that can release antimicrobial agents or absorb ethylene to slow spoilage, as well as intelligent packaging that uses nanosensors to signal freshness or contamination levels.^7–11^ Nanotechnology-based sensors play an important role in the food industry. Because consumers often depend on expiration or sell-by dates rather than direct quality checks, nanosensors can provide real-time information about food condition and help assess freshness more accurately.^10,11^

Nanosensors have applications in food packaging to detect contamination, acting like “noses”.^12^ It is possible to embed nanosensors in or print them on packaging with the purpose of serving as an indication of whether food is fresh or contaminated. For example, a nanosensor embedded in a package of food could change color in response to dangerous bacteria and thus provide a warning signal to both consumers and producers to stop using it.^13^ Such a ‘pre-emptive’ approach increases the safety of food and prevents foodborne diseases. These tools make use of nanomaterials for the identification and monitoring of multiple parameters, including microbial contaminations in different types of assays, chemical residues, and environmental alterations. It is with the help of nanosensors that different types of noxious bacteria, viruses, and fungi are detected in food products. And that is highly important if you think about things like food safety and public health.^14^ They enable you to monitor microbial levels in production areas indefinitely, which means knowing whether hygiene standards are being met or not. Nano-technological sensors can even detect the residues of pesticides and herbicides at very low concentrations, important to know if food complies with safety standards. They will be able to find toxic heavy metals such as lead and mercury present in food items, which is imperative for consumer safety.^15^ Miniature sensors could act as guardians of freshness-by monitoring pH values or temperature, for example, or tracking changes in the levels of volatile organic compounds-that provide an estimate of the quality and shelf life of food.^16,17^ On fruit and vegetables, nanosensors can check for ripeness and condition relating to readiness to eat, thus potentially reducing spoilage rates.^18^ Nanosensors work by interacting with certain kinds of molecules or particles. If the sensor binds to a target, there is a detectable change in, for example, electrical conductivity, optical properties, or mass. This sensitivity translates into real-time surveillance whereby producers and consumers can be alerted quickly if there is a potential problem with food safety.^19^

**Fig. 1:**
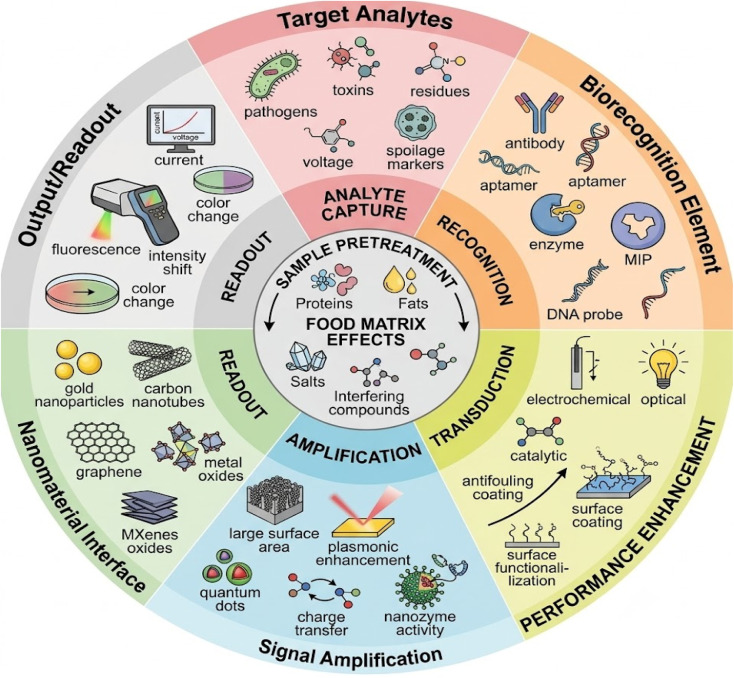
Schematic illustration of nano-sensing principles and nanomaterial design for food analysis (created with https://www.biorender.com/).

Nanosensors are nanoscale devices used in food analysis because they detect substances with very high sensitivity and specificity by combining a recognition element with a transducer that converts binding or reaction events into measurable signals. Electrochemical nanosensors are especially valuable for food testing because they are compact, highly sensitive, and suitable for rapid point-of-need analysis, while optical nanosensors such as colorimetric, fluorescence, and plasmatic systems provide fast visual or instrument-based detection. Nanomaterials including gold nanoparticles, graphene, carbon nanotubes, and quantum dots improve signal amplification and lower detection limits, and surface functionalization helps immobilize bio receptors while improving stability in complex food samples.^20^ In addition, nano-enzymes and catalytic nanomaterials can mimic enzyme activity and strengthen assay signals without the fragility of natural enzymes, although food matrices can still cause fouling and interference, making pre-treatment and antifouling coatings important for reliable results Fig. 1. Recent advances also emphasize multiplexed sensor arrays and pattern recognition for detecting spoilage, adulteration, and contamination, showing that nanosensing and nanomaterial design are driving safer, faster, and more efficient food quality monitoring systems.^2,21^ This review aimed to focus on fundamental research works of use nanosensors for food analysis published in recent years, with special emphasis on their most recent advances.

## 2. Classification and applications of nanotechnology-based food sensors

**Fig. 2:**
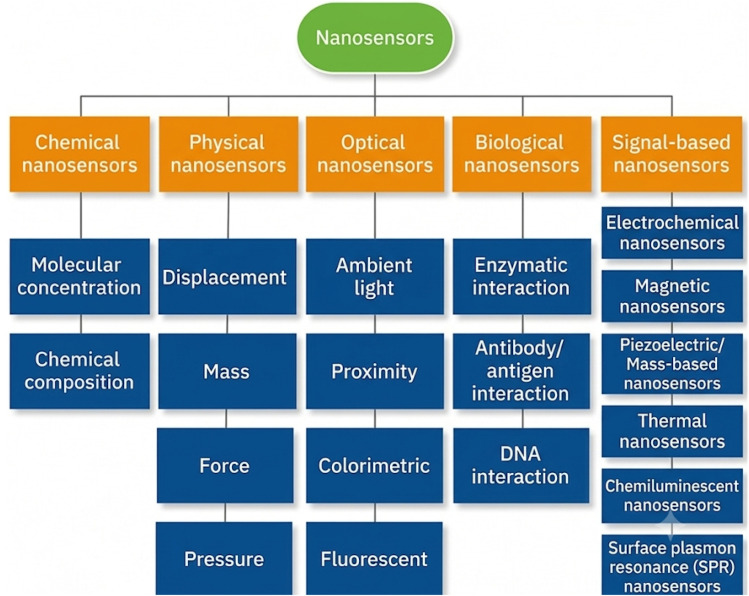
Classified of nanosensors types.^22^ With permission from [Elsevier] [https://doi.org/10.1016/B978-0-12-819870-4.00001-3], copyright 2020.

Nanosensors are minute devices that work in nanoscale dimensions to detect and measure the physical, chemical, optical, biological properties, or signal based nanosensors with high sensitivity and precision. Fig. 2 They can sense at a highly minute scale, usually taking advantage of some unique properties developed in nanomaterials to enable the detection. The most common nanomaterials that offer the ability of these nanosensors include carbon nanotubes, quantum dots, gold nanoparticles, graphene, and metal oxide nanoparticles. Thus, these materials offer definite advantages like high surface area, conductivity, and enhancement in signal transduction.^22^ Nanotechnology-based sensors in food analysis can be classified according to the type of signal they generate or measure, including optical, colorimetric, electrochemical, fluorescent, magnetic, piezoelectric or mass-based, thermal, chemiluminescent, and surface plasmon resonance nanosensors. Optical nanosensors detect changes in fluorescence, absorbance, scattering, or refractive index, while colorimetric nanosensors are a simpler optical type that produces visible color changes in response to target analyzes. Electrochemical nanosensors measure variations in current, voltage, resistance, or impedance, making them highly sensitive for detecting contaminants and biomolecules. Fluorescent nanosensors rely on changes in emission intensity or wavelength, often using nanomaterials such as quantum dots, whereas magnetic nanosensors use magnetic nanoparticles to improve separation and reduce background interference. Piezoelectric or mass-based nanosensors detect changes in mass or mechanical resonance, thermal nanosensors monitor heat-related changes, chemiluminescent nanosensors generate light through chemical reactions, and surface plasmon resonance nanosensors measure shifts in refractive index near nanostructured metal surfaces.

These signal-based classes are supported by nanomaterials such as carbon nanotubes, quantum dots, gold nanoparticles, graphene, and metal oxides, which enhance sensitivity, conductivity, and transduction efficiency in advanced sensing applications.^22,23^ In food analysis, electrochemical nanosensors are commonly used to detect pathogens, pesticide residues, antibiotics, mycotoxins, and nitrites, often achieving detection limits in the nM to pM range or even lower, depending on sensor design.^24^ They are widely applied in milk, meat, fruit juices, and water samples. Optical nanosensors are used for targets such as heavy metals, toxins, pathogens, adulterants, and freshness indicators, with reported detection limits often in the nM to sub-nM range. These sensors are useful for food safety testing, adulterant detection, milk analysis, and freshness monitoring.^25^ Magnetic nanosensors are especially valuable for pathogen and contaminant detection because they can pre-concentrate samples and reduce interference from complex food matrices, making them useful in samples where optical detection is difficult. Overall, nanotechnology-based sensors are being applied to improve food safety, quality control, and freshness monitoring across a wide range of food products.^26^

### 2.1. Physical nanosensors

Physical nanosensors are devices that detect changes in physical parameters such as pressure, temperature, mechanical strain, or vibrations on the nanoscale. Their operation is based essentially on the interaction between the physical stimulus and nanomechanical components like cantilevers, membranes, or nanowires, which respond to physical forces by bending, vibrating, or undergoing strain. The physical change then affects measurable parameters such as electrical resistance, capacitance, or optical signals.^27^ Some physical nanosensors employ plasmonic effects whereby light is confined and enhanced by nanostructures, enabling very sensitive detection through resonance mode changes. Resonance-based physical sensors, such as those using lamb waves (guided waves on thin plates), measure changes in frequency or wave propagation as a function of applied mechanical stress or pressure. The small size provides enhancement of sensitivity *via* an increase in surface-to-volume ratio and by the possibility of quantum mechanical effects that increase the physical interactions.

Applications include structural health monitoring, where small mechanical changes in materials or structures could be measured to prevent failures; environmental sensing for pressure, temperature, and vibrations; and medical applications that use this technology to measure slight physiological changes. Another big application of optical physical nanosensors is for real-time and label-free monitoring, exploiting plasmonics in ultra-sensitive detection. Nano-mechanical sensors are found in precision measurement and also in wearable technology. Thus, in summary, physical nanosensors make use of nanomaterials and mechanisms such as mechanical deformation, resonance, and plasmonic effects to detect minute physical changes with high sensitivity and fast response, with applications in many advanced technological fields. Table 1 compares major physical nanosensor methods and their attainable limits of detection to guide selection according to the needs for sensing and target physical signals.^28^

**Table 1:** Compare of physical nanosensors methods and their limits of detection

Physical nanosensor type	Target analyze	Limit of detection (LOD)	Detection modality	References
Nanomechanical resonators (*e.g.*, cantilever)	Mass, biomolecules, gases	<1 attogram (∼10^−18^ g)	Resonant frequency shift/deflection	28
Surface acoustic wave (SAW) sensors	Gases, biomolecules	∼Parts per billion (ppb) to ppt	Acoustic wave velocity/attenuation	28
Piezoelectric nanosensors	Pressure, force, biomolecules	Piconewton to femtonewton force sensitivity	Piezoelectric signal generation	28
Nanoelectromechanical systems (NEMS)	Mass, force, chemicals	Zeptogram (∼10^−21^ g) range	Resonance frequency shift	29
Nanowire-based physical sensors	Electrical, mechanical quantities	High sensitivity down to single molecule level	Conductance change, mechanical deflection	28
Quantum dots (QD) physical sensors	Temperature, pressure	Sensitivity in micro-Kelvin range	Fluorescence change, optical signals	29
Graphene-based strain sensors	Mechanical strain, pressure	Nanostrain level sensitivity	Electrical resistance change	28

### 2.2. Chemical nanosensors

Chemical nanosensors are devices that detect the presence, concentration, or changes of chemical substances on a nanometer scale. They convert chemical interactions into physically measurable signals such as electrical, optical, or electrochemical responses by utilizing the unique properties of nanomaterials to realize high sensitivity and selectivity. In this regard, there are several types of nanosensors: electrochemical nanosensors, which detect analyzes by measuring current, voltage, or impedance changes caused by chemical reactions at the sensor interface; optical nanosensors use changes in light emission, absorption, or scattering upon analyze binding and include fluorescence and plasmonic sensors. Fiber-optic nanosensors use nanomaterials on optical fibers for real-time, highly sensitive chemical detection. Gas nanosensors detect gases depending on changes in electrical resistance or surface properties of nanomaterials exposed to gaseous analyzes.^27,30,31^ The operation principle of the chemical nanosensors is largely based on the basic fact that a chemical interaction causes a variation in the physical or chemical properties of the sensor's nanomaterial, including its electrical conductivity, fluorescence, capacitance, or surface plasmon resonance. This change is transformed into electrical or optical signals by the transducer component, which is then processed and quantified.

Carbon nanostructures, such as nanotubes and graphene, are commonly used because of their excellent electrical conductivity and large reactive surface area, which enhances signal transduction. Chemical nanosensors based on fiber optics integrate nanomaterials with optical fibers to detect changes in chemicals by changes in light transmission or reflection. Electrochemical nanosensors track changes in charge transfer when analyses are oxidized or reduced at nanoscale electrodes, thereby allowing sensitive detection of gases, ions, and biomolecules.^38^ Chemical nanosensors have a wide range of applications, including environmental monitoring for pollutants and hazardous chemicals, food safety assessment by detecting contaminants or spoilage markers, and medical diagnostics by identifying biomarkers and disease-related chemicals. Table 2 compares the methods of major chemical nanosensor types and their achievable detection limits.

**Table 2:** Comparative limits of detection for chemical nanosensor types

Chemical nanosensor type	Target analyze	Limit of detection (LOD)	Detection modality	References
ZnMn_2_O_4_ nanostructure colorimetric sensor	Omeprazole, lansoprazole	∼0.80–8.8 µg mL^−1^ (∼2–25 µM)	Colorimetric	32
Platinum/palladium nanoparticles on BDD	Hydrogen peroxide	Not quantified but high sensitivity	Electrochemical	33
Poly(luminol-*co*-1,8-diaminonaphthalene)/CeO_2_/MWCNTs	Cr3+ ions	4.8 ± 0.24 pM	Electrochemical	34
Nanoparticle-based electrochemical biosensors	Hormones (cortisol, estradiol, *etc.*)	∼1 nM and lower	Electrochemical	35
Molecularly imprinted polymers (MIPs)	Pesticide residues	Typically low nM to pM	Various (optical, electrochemical)	36
Multi-walled carbon nanotube (MWCNT) based	Aminophenol	9.6 ppb (∼50 nM)	Electrical resistance/pH sensing	37

### 2.3. Optical nanosensors

Optical nanosensors could be defined as devices that detect and measure changes in the intensity, wavelength, polarization, or phase of light resulting from interactions between target molecules and a nanoscale object. They take advantage of unique optical effects within nanomaterials to create devices with high sensitivity and selectivity. Optical nanosensors rely on the variation of light behavior due to the binding of analyze or changes in the environment close to the surface of the sensor. The different mechanisms that support these methods are fluorescence, surface plasmon resonance, Raman scattering, luminescence, and interferometry. Quantum dots, metal nanoparticles, and metal–organic frameworks (MOFs) are examples of many nanomaterials that improve optical signals by plasmonic effects, high fluorescence quantum yields, or multiplexed emission.^39^ In the instance of fiber-optic nanosensors, light is transmitted along optical fibers and is modulated by chemical or biological events occurring along the surface of the fiber, thus enabling remote detection in real time.

Light-induced oscillations of metal nanoparticles are employed in plasmonic nanosensors to monitor minor changes in refractive index, enabling ultrasensitive label-free detection. Luminescence nanosensors monitor the light emitted after excitation of fluorophores, bioluminescence, or chemiluminescence, and they are used in bio-imaging and diagnostics. Medical diagnostics applications include real-time monitoring and the identification of illness biomarkers besides, detection of poisons and contaminants in the environment. To ensure food safety, pollutants and pathogens must be found. Microfluidics integration for diagnostics at the point of care and biological cells and molecules are manipulated and analyzed optically. When combined with quick reaction times, optical nanosensors are often non-invasive, incredibly sensitive, and provide several choices for multiplexed sensing.^40^ Thus, they are quite versatile for biomedical, environmental, and industrial applications. Table 3 compares methods and achievable limits of detection for the major optical nanosensors types.

**Table 3:** Comparative limits of detection for optical nanosensor types

Optical nanosensor type	Target analyze	Limit of detection (LOD)	Detection modality	Reference
Surface plasmon resonance (SPR)	Adenosine	∼0.013–0.018 nM	Optical absorption/plasmonics	41
Fluorescent single-walled carbon nanotubes (SWCNT)	Potassium ions	Physiologically relevant (low nM)	Near-infrared fluorescence	42
Metal oxide nanocomposite (Cr_2_O_3_–TiO_2_)	Hydrogen peroxide	∼0.003 µM	Colorimetric/optical absorbance	43
Gold nanoparticle colorimetric	Tyramine	∼0.014 µM	Colorimetric (plasmonic shift)	44
Polymer passivated fluorescent SWCNT	Interleukin-6 (IL-6)	pg mL^−1^ range (∼picomolar)	Near-infrared fluorescence	45
Nanogap plasmonic biosensors	miRNA biomarker	∼0.78 nM	Plasmonic resonance	46
Optical microcavity with DNA nanostructures	DNA/biomolecules	Ultra-low (1000x traditional)	Resonance fluorescence enhancement	47
Aptamer-QD-gold nanoparticle FRET sensor	C-reactive protein	∼1.77 pM	Fluorescence resonance energy transfer (FRET)	48
Heteroatom-doped carbon dots	Glutathione (GSH)	∼80 nM	Fluorescence “on-off-on”	49
MOF-based fluorescent nanosensor	Hg2+ ions	8.23 × 10^−8^ M (0.0823 nM)	Fluorescence	50

### 2.4. Magnetic nanosensors types

The principle of magnetic nanosensors relies on the detection of targets by magnetic nanoparticles, beads, or quantum defects with very high sensitivity, from nanomolar to attomolar concentrations, depending on the system. They allow for sensitive, specific, and often label-free detection in environmental monitoring (pollutants, toxins), biomedical diagnostics (early disease markers detection, cancer biomarkers), food quality control-pathogens and contaminants detection, and agricultural monitoring-soil nutrients, pesticides.^51,52^Table 4 summarizes in detail their limits of detection and working principles, presenting the choice of appropriate magnetic nanosensors technologies in various applications.

**Table 4:** Comparative limits of detection, working principle, and advantages for magnetic nanosensor types

Magnetic nanosensor type	Working principle	Limit of detection (LOD)	Advantages	References
Magnetic nanoparticle-based fluorescent nanosensor	Fluorescence quenching/enhancement *via* magnetic nanoparticles combined with polymer dots or COFs	∼1.2 nM (imidacloprid)	High sensitivity, sample preconcentration, matrix interference reduction	52
Magnetic bead-based SERS nanosensor	Ratiometric surface-enhanced Raman scattering with magnetic beads for target capture	∼2.067 ng mL^−1^ (matrix metalloproteinase 2)	Ultrasensitive, specific, internal calibration for quantification	53
Up conversion magnetic nanosensor	Magnetic separation combined with up conversion luminescence and amplification	∼0.093 aM (extracellular ARGs detection)	Ultra-sensitive, rapid detection, good selectivity	54
Magnetic nanoparticle-based electrochemical sensors	Magnetic nano-structured sensors with magneto resistive detection	Single particles or ∼ sub-nanomolar range	Ultrasensitive, label-free, rapid response	53
Magnetoelastic immunosensor	Magnetic nanoparticles amplify mechanical resonance changes upon analyze binding	< 1 nM (human IgG detection)	Wireless, real-time, sensitive	55
Nitrogen vacancy (NV) centers in diamond	Quantum sensor detecting nanoscale magnetic fields with high sensitivity	∼0.5 nT/√Hz (magnetic field sensitivity)	Nanoscale spatial resolution, highly sensitive	56

### 2.5. Electrochemical nanosensors

Electrochemical nanosensors represent one of the leading biosensing platforms that combine nanotechnology with electrochemistry to attain ultrasensitive detection of a wide variety of chemical and biological molecules. The high sensitivity of the electrochemical nanosensors is due to the large reactive surface area of nanomaterials. Their adaptability and high performance make them increasingly important in health monitoring, environmental analysis, biosecurity, and common in detecting gases, ions, and biomolecules.^57^Table 5 displayed the comprehensive multiple recent advances in sensor fabrication, functional nanomaterials, and electrochemical nanosensor detection strategies for various applications.

**Table 5:** Compare biosensing transduction methods and their limits of detection with working principles

Transduction method	Working principle	Limit of detection (LOD) and limitations	Advantages	References
Electro-chemical	Measures changes in current, voltage, impedance, or conductance caused by binding events. Applications include voltametric, potentiometric, impedimetric, and FET-based sensors	Typically pico- to nanomolar (pM to nM). Susceptible to interference, requires electrode modification, limited multiplexing	High sensitivity, simple instrumentation, miniaturizable, cost-effective	28
Optical	Detectors of changes in the properties of light resulting from the binding of a target, which includes fluorescence biosensors, SPR, and photonic sensors	Femtomolar (fM) to nanomolar (nM). Complex optics setup, costly, sometimes requires labeling	Label-free detection, fast response, high specificity, multiplexing	58
Mechanical (piezoelectric, cantilever)	It detects changes in mass or mechanics stress, resonance frequency shifts on nanoscale resonators or cantilevers due to the binding of analyzes	Nanomolar to micromolar range. Sensitive to environmental noise, complex fabrication, sometimes slower response	Label-free, real-time measurements, high sensitivity	59
Electrical (FET-based)	Detects changes in conductance or resistance at nanoscale transistor channels upon molecular interactions. It involves nanomaterials such as graphene or carbon nanotubes	Pico- to nanomolar levels. Complex fabrication, potential signal drift, surface fouling	Ultra-sensitive, real-time detection, easy integration with electronics	58

## 3. Nanosensors benefits in various applications

### 3.1. High sensitivity

Nanosensors are highly sensitive primarily due to their unique nanostructured materials, which, with a high surface-to-volume ratio, increase the interaction with the target molecules. Large active surface areas allow for more binding sites, increasing the possibility of detecting even very low concentrations of analyzes. Secondly, nanomaterials enhance electrical, optical, and mechanical signal transduction to amplify sensor response.^28^ Such mechanisms include reduced charge screening in a nanostructured electrode, accelerating electron transfer and boosting sensitivity, and biomimetic designs amplifying mechanical interactions. Other structural features that enhance surface area include 3D nanowalls, which further improve performance.^60,61^ Such properties enable nanosensors to detect biomarkers, chemicals, or pollutants at ultralow concentrations with fast response times, often at the nanoscale level.

### 3.2. Fast response in real-time monitoring applications

Nanosensors respond quickly because they are very small and have a high surface-to-volume ratio, which lets them quickly adsorb and interact with target molecules. Their nanostructured materials let electrical, optical, or mechanical signals change quickly when molecules bind to them. Combining with materials like metal oxides or fluorescent probes that are trapped in Nano channels makes it possible to quickly and reversibly detect things, often in less than minute or even milliseconds. Also, nanowire-based sensors and 2D Nano mechanical resonators have very fast intrinsic response times because of improved charge transfer kinetics and mechanical vibrations. This makes it possible to monitor gases, biomolecules, or environmental stimuli in real time.^62–64^ This fast transduction process is very important for uses that need quick detection, like finding hydrogen gas leaks or viral RNA, or temperature monitoring.

### 3.3. Small size and portability

Nanosensors have a number of advantages because their small size and, therefore, easy portability allow miniaturization into compact and portable devices suitable for point-of-care medical diagnosis, environmental monitoring, and food safety. Devices such as this can be used in areas that are either distant or lack sufficient resources to quickly detect something with ease.^65^ Due to their minuscule size, nanoscale sensors are able to provide health monitoring capability without invasion into the human body.^66^ Being of small size, it can be integrated with microfluidic and other lab-on-a-chip systems to create automated, rapid, and high-throughput analytical platforms.^67^ The portability and user-friendly performance enable non-professional users to conduct complex analyses out of conventional laboratories, facilitating access to timely, and personalized diagnostic information. Generally, low power consumption and material costs of nanosensors foster the development of disposable or single-use sensors.^68^ Thus, the combination of small size and portability allows nanosensors to be versatile tools that improve real-time monitoring capabilities with high sensitivity and speed in various applications, from clinical diagnostics up to environmental surveillance. This contributes to their huge possible impact upon healthcare, food safety, and environmental monitoring due to the possibility of the provision of fast, accessible, and precise sensing solutions.

### 3.4. Selectivity and specificity of nanosensors

**Fig. 3:**
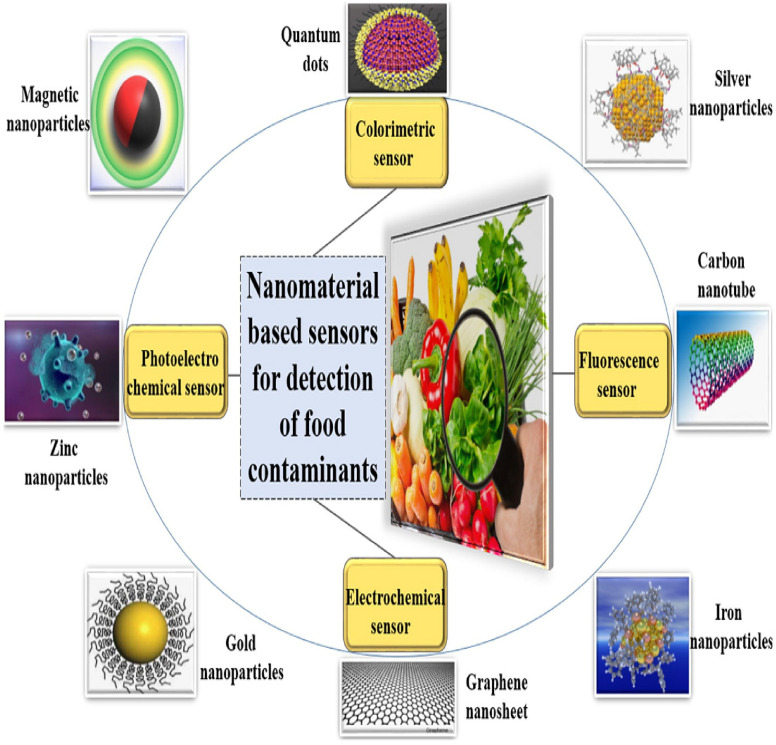
Nanomaterial based different sensors for detection of food contamination.^74^ with permission from [Taylor & Francis] [https://doi.org/10.1080/28361466.2024.2373196], copyright 2026.

Nanosensors reach high selectivity and specificity mainly through the integration with the selective receptors, including antibodies, peptides, enzymes, and synthetic receptors binding exclusively to target analyzes. The receptors are stuck to the nanosensor surface, which lets it tell the difference between target molecules and other similar or interfering molecules. To enhance selective binding, people often operate with surface functionalization techniques and catalyst decoration. Nanomaterials, including gold, zinc and silver nanoparticles, quantum dots, graphene nanocheet, and carbon nanotubes, not only augment signal amplification but also function as carriers for recognition elements, thus improving overall detection accuracy, especially in respect to food safety monitoring, as showing in Fig. 3.^69–71^ Advanced design strategies involve multiplex detection and integrate machine learning, enhancing selectivity by facilitating simultaneous detection of various targets and the resolution of complex sample mixtures. This capability is critical mainly for the detection of food contamination, early diagnosis of cancer, and detection of environmental pollutants.^72,73^ The combination of these molecular and nanostructured methods allows nanosensors to provide reliable, target-specific responses even in complex biological or chemical environments.

### 3.5. Versatility in applications

Nanosensors have remarkable adaptability in applications across several sectors due to their adjustable optical, electrical, and electrochemical capabilities. These are applied to biomedical sensing for the detection of pathogens, early disease diagnosis, and the monitoring of physiological parameters. Nanosensors are used in environmental monitoring for the sensitive identification and detection of hazardous materials and pollutants.^75,76^ The food industry uses them for ensuring the safety and quality of the food. More importantly, nano-sensors are integrated with wearable and implantable devices, which monitor health in real time. Compatibility with portable platforms and machine learning enhances their versatility for prompt point-of-need detection in challenging environments. In general, nanosensors are practical and useful tools in environmental science and agriculture, healthcare, among others, because their nanomaterials and sensor mechanisms can be suitably adapted to a range of specific analyses and environments.^77,78^

### 3.6. Enhanced signal transduction

Enhanced signal transduction mechanisms in nanosensors are obtained by using unique properties of nanomaterials that amplify the detection signal produced after the interaction of target analyzes. The nanomaterials used, which include semiconductor nanowires, carbon nanotubes, gold nanoparticles, and quantum dots, result in more intensive electrical, optical, electrochemical, and mechanical output signals. They provide a large surface area and, at the same time, supply efficient charge transfer pathways. The advanced mechanisms include mechanical transduction in Nano-mechanical resonators, electrochemical signal amplification *via* accelerated electron transfer, and optical transduction *via* fluorescence and plasmonics.^28^ High sensitivity and selectivity with enhanced outputs are attained by incorporating molecular recognition components into nanostructures. In addition, richer data and accuracy are attained in multimodal nanosensors due to the integration of several synergistic transduction mechanisms. This improved signal transduction provides a chance for the detection of incredibly low analyzer concentrations in biomedical diagnostics, environmental monitoring, and food safety with fast reaction times.^79,80^

### 3.7. Potential for integration

Nanosensors can be integrated with machine learning and data analysis techniques for advanced detection platforms. They can be integrated into wearable, implantable, and portable devices for real-time health monitoring and diagnostics. Integration with telemedicine and digital health platforms enables personalized healthcare and remote patient monitoring, enhancing efficiency and access. In addition, nanosensors can be integrated into Internet of Things (IoT) systems used for industrial process control, agriculture, and environmental monitoring, thus offering constant data streams that allow for astute decision-making.^81,82^ The integration of machine learning and artificial intelligence into the nanosensor further enhances its performance by improving data analysis sensitivity and multiplexing. The development of microfluidics and miniaturized electronics allows integration of nanosensors into lab-on-a-chip technologies for rapid on-site analysis. Because of these integration opportunities, nanosensors are very versatile for a wide range of applications such as food safety, environmental monitoring, and healthcare, among many others.^78,83^

### 3.8. Noninvasive and continuous monitoring

Nanosensors enable noninvasive and continuous monitoring through various advanced techniques and device designs. Given their ultra-small size and biocompatibility, wearable or implantable platforms can integrate into them for real-time physiological monitoring without the use of invasive procedures.^84,85^ Non-invasive monitoring makes use of biofluids such as sweat, saliva, tears, and interstitial fluid to continuously and painlessly monitor biomarkers such as glucose, electrolytes, and metabolites. The various methods to detect changes in these biofluids or tissue properties include optical, electrochemical, and acoustic sensing techniques. Examples of wearable devices which maintain skin contact for continuous health monitoring include microneedle arrays, skin patches, smart textiles, and flexible electronics. These parameters range from blood pressure and haemoglobin to glucose levels to hydration. The interpretation of results for quick diagnostics is enhanced through integration with AI and machine learning. Continuous monitoring nanosensors would bring a new level of convenience and accessibility to real-time health tracking in specific medicine, remote healthcare, and, importantly, the management of chronic diseases.^86–88^

## 4. Comparative performance, limitations, and validation challenges of nanosensors in food analysis

Nanosensors for food analysis have shown strong promise because they offer high sensitivity, rapid response, and miniaturized detection, but their performance must be evaluated beyond basic detection capability. Electrochemical, optical, and magnetic nanosensor platforms each provide distinct advantages and trade-offs. Electrochemical nanosensors, such as those based on gold nanoparticles and carbon nanotubes, can achieve ultralow detection limits and fast response times, yet they are vulnerable to electrode fouling by food proteins and lipids and can be strongly affected by pH and temperature fluctuations. Optical nanosensors, including quantum dots and plasmonic nanoparticles, enable real-time, multiplexed detection through distinct spectral signatures, but they may suffer from photobleaching, reduced sensitivity in turbid or colored food matrices such as wine and juice, and batch-to-batch variation in nanoparticle size that shifts plasmonic peaks. Magnetic nanosensors, such as iron oxide nanoparticle systems, are more resistant to optical interference and can support large-volume sample pre-concentration, but they often require bulky detection equipment, longer incubation and washing steps, and offer lower spatial resolution.^13^

Across all nanosensor types, poor colloidal stability remains a major limitation, as nanoparticles can aggregate in high-salt or acidic foods or form protein coronas that block active surfaces, leading to signal degradation over time. Reproducibility is also a critical issue because small differences in nanoparticle size, synthesis conditions, or surface chemistry can cause meaningful variation in optical and electrochemical signals. Matrix effects further complicate analysis, since pigments, lipids, salts, and enzymes in complex foods can absorb light, coat sensor surfaces, degrade biorecognition elements, or suppress signals, producing false positives, false negatives, or reduced analytical precision. For this reason, sample pretreatment, antifouling coatings, and matrix-matched calibration are often necessary.^13^ Validation quality is another major concern, because many published nanosensors are tested only under controlled laboratory conditions rather than against established reference methods; robust validation should therefore include accuracy, precision, recovery, linearity, specificity, limit of detection, and comparison with standard techniques such as ELISA, PCR, or chromatography.^89^

Scalability and commercialization also remain limited because of fabrication complexity, batch-to-batch variation, storage instability, and the need for specialized instrumentation or trained personnel.^13^ In addition, no current method has yet achieved truly industrial-scale production of monodisperse, functionalized nanoparticles for routine food testing, and regulatory frameworks still do not fully address nanomaterial-specific issues such as leaching, long-term stability, or toxicity. Emerging solutions, including microfluidic automation, machine-learning-based drift correction, silica encapsulation, and internal Raman normalization, are promising but remain largely at the research stage.^21^ Overall, the most promising nanosensors are those that combine strong analytical performance with practical durability, simple operation, reliable testing in real food samples and compatibility with standardized validation pathways.

In spite of rapid growth, practical deployment of nanosensors in food systems is constrained by matrix complexity, incomplete standardization, and barriers to scale-up and regulation. Future work should prioritize matrix-resilient designs, combined validation protocols, low-cost in addition to scalable fabrication, besides integration with smart packaging and digital traceability systems to enable safe, sustainable, and commercially viable deployment.

## 5. Recent applications of sensors in the food industry

Smart IoT-enabled sensors in packaging monitor variables such as humidity and temperature to prevent food spoilage. Methods such as Fourier Transform Infrared (FTIR) spectroscopy and Near Infrared (NIR) spectroscopy enable rapid on-site quality testing. Electrochemical sensors, including time–temperature indicators (TTIs), track temperature exposure during transport. Foodborne infections and contaminants are diagnosed with nanosensors, many of which employ machine learning for enhanced detection. Hyperspectral imaging and Raman spectroscopy monitor food quality in real time while RFID and NFC technology enhance traceability and compliance with regulatory requirements. Thermochromic labels on smart packaging bring spoiling risks to the fore. Working together, these technologies address food safety, quality control, and sustainability throughout the value chain.^90^Table 6 describes current advancements and prevailing sensor applications that are crucial for food safety, quality, and traceability within the food industry.

**Table 6:** Overview of common sensor technologies in modern food processing

Sensor application	Food industry use	Examples	Reference
IoT-enabled biosensors in packaging	Real-time monitoring of food freshness, spoilage, pathogen detection; integrated with IoT for data transmission and decision support	Electrochemical biosensors embedded in packaging; monitor temperature, humidity, pH, contamination	91
Gas sensors	Detect freshness and spoilage gases (CO2, ethylene, rancidity indicators); pesticide detection	Electrochemical, MOS sensors, IR absorption methods	92
Temperature and humidity sensors	Crucial for controlling storage and transport conditions to maintain food safety and quality	Thermocouples, IR sensors, capacitive humidity sensors	91
Biosensors	Detection of pathogens, allergens, toxins for food safety	Enzyme-linked, antibody and DNA biosensors	91
Smart packaging and thermochromic labels	Visual indication of temperature abuses and quality deterioration during transportation and storage	Color-changing smart labels, temperature/time indicators	92
AI-powered sensor systems	Data analytics for quality control, spoilage prediction, traceability	Integration of sensor data with AI and blockchain for supply chain transparency	93
Optical sensors	Non-destructive quality attributes evaluation such as color, texture, and foreign material detection	Raman spectroscopy, NIR, colorimetric sensors	94
RFID and GPS tracking	Supply chain monitoring to ensure freshness and reduce counterfeiting	RFID tags, GPS sensors integrated with real-time monitoring	91
Ultrasonic and metal detection	Internal defect inspection and contaminant detection	Ultrasound imaging, industrial metal detectors	93

### 5.1. Applications of nanosensor on different milk and cheese types

A variety of nanosensor platforms, such as enhanced infrared absorption, colorimetric paper strips, deep learning image-based systems, plasmonic dual-mode sensors, and IoT-integrated impedance sensors, have been employed for the analysis of various milk types, including cow, camel, goat, buffalo, and infant formula. These approaches advance milk quality and safety because they enable the rapid and sensitive field detection of various adulterants, including hydrogen peroxide, lactic acid, urea, ammonium sulphate, oils, and synthetic additives.^95^ Recent studies of milk adulterant detection, described in (Table 7), highlight the use of various nanosensor types, including colorimetric strips and machine learning-enhanced image sensors, which have limits of detection that equal or better many regulatory thresholds. These tools are crucial to milk safety.

**Table 7:** Recent nanosensor applications on milk adulteration detection

Milk samples	Nanosensor type	Target analyses	Limit of detection (LOD)	Applications	References
Liquid cow milk	Citrate-capped silver nanoparticle-enhanced SEIRA sensor	Adulterants and milk constituents	Qualitative and quantitative detection (high sensitivity)	Differentiation of cow, camel, goat, buffalo, infant formula powdered milk	95
General milk types (raw &processed milk)	Zein/MnO_2_ nanosheet composite colorimetric sensor	Hydrogen peroxide, lactic acid adulterants	LOD ∼7.2 × 10^−4^ mol L^−1^ (H_2_O_2_) and ∼7.5 × 10^−4^ mol L^−1^ (lactic acid)	Rapid, on-site, dual detection of adulterants	96
Various types of liquid milk samples	CNN-based image sensor (evaporative deposit patterns)	Urea, ammonium sulfate, oils adulterants	98% detection accuracy	Effective classification and quantification of multiple adulterants	97
Liquid milk (general)	Gold nano-bipyramids LSPR dual-mode sensor	Urea adulteration	LOD 0.09 µM (colorimetric), 0.056 µM (fluorometric)	Highly sensitive colorimetric on-site monitoring of urea adulteration	98
Liquid milk (various types)	Fractional-order impedance sensor	Synthetic milk and adulteration	High accuracy and specificity	Differentiation of fake and real milk using impedance and IoT integration	99
Milk (fresh to spoiled)	Silver nanoparticle colorimetric nanosensor	Lactic acid (spoilage marker)	LOD 5 mL L^−1^ for lactic acid	Rapid, sensitive detection of milk spoilage at pre-spoilage stage	100
Milk samples	Electro-chemical + spectroscopic sensor	Various adulterants	High accuracy, real-time detection	Portable, low-cost real-time detection of milk adulteration	101
Milk samples	Microfluidic paper-based nanosensor	Multiple adulterants (urea, detergents, salt, *etc.*)	Simultaneous multi-adulterant detection	First simultaneous multi-adulterant detection device, improving over single-target tests	102
Pure milk powder	Electronic nose equipped with 8 metal oxide sensors	Detect melamine adulteration	High accuracy, effective on dry/wet and pure/adulterated milk samples	Rapid detection and classification of pure milk powder from adulterated materials	103
Milk powder	Metal oxide semi-conductor (MOS) sensors MOS electronic nose	Detect whey adulteration in powdered milk	Sensor response varies with wet/dry samples, 95.6% detection accuracy	Rapid quality assessment of commercial powdered milk, improving discrimination	104

Similarly, nanosensors in cheese are represented by electrochemical sensors capable of microbial contamination and pesticide residues, and fragrance and gas analysis for quality control. All these sensors possess very low limits of detection to enable improved food safety and quality verification. Sample matrices, cheese kinds, nanosensor types, and LODs reported in recent studies are summarized in (Table 8).

**Table 8:** Nanosensor applications studies on cheese adulteration detection

Cheese type	Nanosensor type	Target analyzes	Limit of detection (LOD)	Applications	References
Roquefort, blue stilton, blue cheese spread	Chromogenic array/Optoelectronic nose	Aroma volatile compounds	Not explicitly reported	Accurate classification and food fraud detection *via* aroma fingerprints	105
Parmigiano reggiano	S3 nanowire gas sensor	Rind content, aroma gases	0.27 ppb (LOD for cheese aroma volatiles)	Quality control through rind percentage identification	106
Various cheese types	Electro-chemical nanosensors/Biosensors	Pathogens, toxins	Sensitivity down to few CFU mL^−1^ for pathogens	Rapid and sensitive microbial safety monitoring	107
Processed cheese	Enzyme-based electrochemical sensors	Organophosphorus pesticides	Detection limit as low as 3 × 10^−12^ mol L^−1^ (sub-nM range)	Ultra-sensitive detection of pesticide residues	108
Grated hard cheese	Optical nanosensor near-infrared (NIR) spectroscopy	Adulterants (cellulose, wheat flour, silicon dioxide)	1.96–7% (w/w)	Detection of adulterants and authentication of PDO cheeses	109
Various cheese types	Nuclear magnetic resonance (NMR) nanosensor	Lipid and aqueous biomarkers	Not explicitly stated	Differentiation of protected designation of origin (PDO) cheeses	109
Cheese samples	Mass spectrometry nanosensors	Protein-based adulterants, species-specific peptides	Trace level quantification	Sensitive detection of adulterants and species-specific identification	110
Cheese matrices	Electrochemical biosensors with nanosensor	Foodborne pathogens and toxins	Down to a few CFU mL^−1^ (bacteria)	Rapid, sensitive detection and improved food safety	107

### 5.2. Applications of nanosensor on different meat and fish types

In the food industry, especially regarding different types of meat and fish, nanosensors have become advanced tools for analysis. These nanoscale devices integrate biological recognition elements with transducers, enabling the fast and highly sensitive detection of target substances that include pathogens, toxins, and chemical contaminants. Their utilization in meat and fish products facilitates real-time monitoring of freshness, quality, and safety, allowing for early detection of spoilage and contamination that lowers health risks due to foodborne illness and aids in compliance with regulatory issues within the food supply chain. Nanosensors improve nutritional value detection, adulteration, and freshness indicators in meat. Nanosensors are one of the important tools in the current quality control and assurance of safety in meats and fish because they utilize nanomaterials such as graphene, carbon nanotubes, and metallic nanoparticles that greatly enhance sensitivity, specificity, and stability of signals^21,111^ illustrate the use of nanosensors on various kinds of meat and fish, respectively, based on current research (Tables 9 and 10).

**Table 9:** Nanosensors applications on different types of meat

Meat sample	Nanosensor type	Target analyzes	Limit of detection (LOD)	Applications	References
Pork (meat extracts)	Optical biosensors (hyperspectral imaging)	Species classification	Not explicitly stated	High accuracy (∼95%) portable meat species authentication	112
Beef (fresh meat samples)	Electro-chemical biosensors	Purine metabolites (xanthine, hypoxanthine)	LOD 0.01 to 58 nM for xanthine	Effective freshness assessment with rapid response	113
Chicken (raw meat & processed)	Electro-chemical biosensors	Bacterial contamination, spoilage markers	Low nM to µM levels	Rapid microbial and freshness detection	113
Lamb meat tissue	Optical biosensors, DNA biosensors	Species identification, microbial toxins	Not explicitly stated	Accurate species authentication and toxin monitoring	114
Mixed meat products	DNA-based biosensors	Meat species identification	Very low detection limits with DNA amplification	Reliable detection of adulteration in complex meat mixtures	115
Fresh chicken meat	Colorimetric nanosensor using silver nanoparticles + smartphone imaging	Spoilage VOCs (volatile organic compounds)	Not explicitly reported	Rapid detection of chicken meat spoilage with high accuracy	116
Chicken skin and meat	Carbon nanotube (CNT)-based electrochemical nanosensor	Salmonella bacteria	Few CFU mL^−1^	Sensitive detection of Salmonella contamination	21
Chicken meat	Silica nanoparticle enhanced DNA dot blot biosensor	Campylobacterspp. DNA	As low as 10^2 CFU mL^−1^	Highly sensitive detection of Campylobacter in chicken meat	117
Chicken meat	Gas sensor nanosensor array	Volatile compounds fromE. Coli	Not specified	Classification of bacterial contamination on chicken	118
Chicken meat	Optical biosensor SPR (surface plasmon resonance) biosensors	Allergen proteins, pathogens	Low nM concentration	Label-free real-time detection for food safety applications	111
Beef tissue extracts	Nanomaterial-enhanced electrochemical biosensor	Biogenic amines (freshness markers)	Low nanomolar range	Sensitive detection of spoilage indicators for freshness assessment	111
Cow meat samples	Conductive nano-silver ink electrochemical sensor	Histamine (spoilage indicator)	10 nM (lower quantification limit)	Rapid, reproducible, affordable histamine detection to monitor meat quality	119
Beef samples	Electronic nose (sensor array)	Odor volatile compounds (freshness)	Not explicitly reported	Automated discrimination between fresh and stale beef	120
Beef and derived products	Fluorescent nanobiosensors	Pathogenic bacteria (*e.g.*, *E. coli*)	Down to 6 CFU mL^−1^ (bacterial cells)	Rapid pathogen detection in complex food matrices	121
Meat mixtures (duck, beef, pork)	DNA amplification + multiplex lateral flow nanosensor	Duck DNA and multiple species	10^1^ copies per 50 µL reaction	Rapid 20 min onsite detection of duck, pork, and bovine DNA; detection of 0.1% adulteration	122
Processed duck meat	Fluorescent nanosensor	Duck-specific DNA sequences	Not explicitly stated	High sensitivity detection of adulterated duck meat	123
Minced lamp meat with duck adulteration	Optical nanosensor	Adulteration detection	Not explicitly stated	Non-destructive, rapid detection of duck adulteration in lamb	124
Meat products (chicken, duck, goose)	Multiplex PCR nanosensor	Species DNA	Low detection limits enabled by multiplex PCR	Simultaneous detection of multiple poultry species in meat products	125
Duck meat	Metabolomic nanosensors + genome-wide assay	Meat quality markers	Not explicitly stated	Novel genetic and biochemical insights into duck meat quality	126
Raw and processed camel meat	DNA-based PCR nanosensor targeting mitochondrial cytochrome b gene	Camel meat DNA	Absolute LOD ∼10 pg, relative LOD ∼45 pg	High specificity, no cross-reactivity to cattle, buffalo, sheep, goat, chicken, pork	127
Camel meat (adulterated samples)	Fluorescent sensing platform with Zr-MOF magnetic nanocomposite nanosensor	Pork adulteration in camel meat	Not explicitly reported	Effective identification of pork adulteration in camel meat	128
Camel meat samples	PCR-RFLP based nanosensor	Species differentiation (camel *vs.* others)	Low pg-level PCR amplified DNA	Cost-effective, rapid verification of camel meat authenticity	127

**Table 10:** Nanosensor applications on different types of fish

Fish types	Nanosensor type	Target analyzes	Limit of detection (LOD)	Applications	References
Olive flounder (*Paralichthys olivaceus*)	Enzyme nanosheet electrochemical biosensor	Aspartate (biomarker for bacterial infection)	500 µM	Enables point-of-care rapid bacterial infection diagnosis	129
Gilthead seabream (*Sparus aurata*)	Implantable near-infrared fluorescent nanosensor hydrogel	Riboflavin (oxidative phosphory-lation marker)	Not specified	Real-time physiological biologging without affecting behavior	130
Catshark (*Galeus melastomus*) and other shark species	Implantable fluorescence nanosensor hydrogel	Riboflavin	Not specified	Monitoring sensor design parameters, biocompatibility	130
Sarasa comet goldfish (*Carassius auratus*)	Near-infrared fluorescent nanosensor	Riboflavin	Not specified	No adverse impact on swimming or feeding behavior	130
Fish nervous necrosis virus (nod virus)	Gold nanoparticle-based lateral flow biosensor	Viral nucleic acids	Not stated	Visual detection of viral pathogens in fish tissue	131
Fish muscle tissue	Mass spectrometry-based nanosensor	Fish species biomarkers	Not specified (high sensitivity)	Rapid species differentiation with ∼99% accuracy	115
Fish muscle extract	Capillary electrophoretic nanosensor	Parvalbumin allergen	0.71 µg mL^−1^	Fast allergen detection in fish samples	132
Fish tissue	Mass spec nanosensor	Pesticides	Low ng g^−1^ range	Broad screening for trace pesticide residues	133
Fish muscle tissue	LC-MS nanosensor	Organic pollutants	Low µg kg^−1^ to ng kg^−1^ range	Sensitive detection of contaminants in fish products	134

### 5.3. Nanosensor applications on different types of fruits and vegetables

The fruit and vegetable sector uses nanosensors as innovative instruments to monitor freshness, safety, and quality along the whole supply chain. These nanoscale sensors exhibit very good sensitivity and specificity for detecting key factors in ripeness, microbial contamination, nutritional levels, and environmental factors such as temperature and humidity. Putting nanosensors either in packaging or on produce allows the tracking of maturity phases and spoilage signs such as ethylene gas in real time, which helps reduce food waste and ensures that consumers get fresh and safe products. Moreover, nanosensors aid in better post-harvest management and enhance shelf-life by permitting timely interventions. The addition of nanomaterials such as silver nanoparticles enhances the performance of nanosensors, thereby proving their potential applicability in biochemical and environmental sensing in fruits and vegetables. This advanced technology promotes sustainable practices and increases customer trust in the quality of food.^135,136^ Nanosensor applications on several fruit and vegetable varieties were established in Tables 11 and 12, respectively.

**Table 11:** Nanosensor applications on different types of fruits

Fruit type	Nanosensor type	Target analyzes	Limit of detection (LOD)	Application	References
Apples, berries (fruit surfaces)	Silver nanoparticle-based SERS sensors	Pesticides (*e.g.*, organo-phosphates)	Sub-ppb levels	Rapid pesticide detection in under 5 min, food safety use	137
Oranges, straw-berries, kiwifruit	CoOOH@BSA-FITC fluorescent nanosensor	Vitamin C (ascorbic acid)	Visual detection at minimal conc	Fast, portable freshness and nutrient testing	138
Fruit plants (citrus, potatoes)	Optical immunosensors (LFIA, lateral flow assays)	Tomato mosaic virus, citrus tristeza virus	2 ng mL^−1^	Sensitive, rapid detection, early disease intervention	139
Fruit juices (various)	Chemometric-enhanced sensor arrays	Adulterants (sugar, syrup, water)	Not specified	Accurate adulteration detection, authenticity assurance	140
Fruit juices (orange, apple)	Gold nanoparticle-based colorimetric sensors	Authenticity markers, adulterants	pM to nM levels	Rapid, portable testing for juice adulteration	141
Apple surface	Optical nanosensor (SERS-based)	Pesticide residues (parathion-ethyl)	Detection within 5 minutes; low concentrations detected	Rapid, non-destructive pesticide residue detection on apples	142
Apple peel	Seed-mediated TMA-Ag/AuNPs SERS substrate	Thiram pesticide	0.258 ppb	High sensitivity, selectivity, excellent recovery, food safety enhancement	143
Apple extracts	DNA nanosensor	Organophosphorus pesticides	Trace level detection	Environmental monitoring of pesticide residues on apples	140
Whole apple images	Imaging nanosensor (optical)	Ripeness, quality indicators	Not specified	Enhanced quality and ripeness detection accuracy	141
Fruit surface and juice	Fluorescent quantum dot nanosensor	Heavy metals (*e.g.*, Pb, Cd)	Low nmol L^−1^ range	Fast, highly sensitive detection of heavy metal contaminants	14
Fruit tissue	Nanopore sensor (oxford MinION)	Plant pathogen DNA	Comparable to PCR (fg level)	Real-time identification of fruit pathogens (*e.g.* citrus)	144
Whole fruit (postharvest)	Flexible optical nanosensor	Fruit ripeness and freshness	Not explicitly quantified	Real-time, non-destructive quality monitoring in cold chain	145
Various fruit tissues	Colorimetric lateral flow assay	Viral plant pathogens	pg mL^−1^ level	Rapid visual detection of viral diseases in fruits	139
Fruit packaging	Nanomaterials for detecting gases and microbes	Gases indicating spoilage	Not specified	Advanced packaging and spoilage prevention *via* nanosensor detection	92
Banana fruit	Multiwalled carbon nanotube (MWCNT) sensor for ethylene gas	Ethylene gas (ripening indicator)	Low concentration ethylene detectable	Early, *in situ* spoilage detection *via* ethylene monitoring, preventing food waste	146
Mixed fruits and vegetables	Array of MQ gas sensors with AI	Spoilage-associated gases	Not specified	Real-time monitoring of gas emissions, humidity, temperature for spoilage prediction	147
Packaged fruits	Nanofiber taper with functional metal oxide coatings	Volatile organic compounds (VOCs), ammonia	High sensitivity	Real-time food quality and spoilage monitoring, freshness preservation	14

**Table 12:** Nanosensor applications on different types of vegetables

Vegetabels type	Nanosensor type	Target analyzes	Limit of detection (LOD)	Application	References
Various vegetables	Multiwalled carbon nanotube (MWCNT) sensor for spoilage gases	Ethylene gas, VOCs associated with spoilage	Low gases detected	*In situ*, real-time spoilage a monitoring to reduce food waste	146
Tomato-based vegetables	Array of MQ gas sensors integrated with AI algorithms	Spoilage gases, humidity, temperature	Not specified	Real-time gas emission monitoring and spoilage classification	147
Tomato fruits	Electro-chemical biosensor	Pesticides (*e.g.*, 4-nitrophenol)	ng mL^−1^ range	Effective pesticide residue detection for food safety	139
Packaged vegetables	Nanofiber tapers with metal oxide coatings for VOCs and ammonia	Volatile organic compounds (VOCs), ammonia	High sensitivity sensor	Quality and spoilage monitoring during packaging and storage	14
Fresh vegetables	Nanomaterial sensor arrays for gases and microbial detection	Spoilage gases, microbial toxins	Not specified	Enhanced shelf-life monitoring and real-time spoilage alerts	92
Vegetables in cold chain	Chemosensors based on nanomaterials	VOCs, ammonia (spoilage markers)	Trace detection possible	Detection of spoilage onset in cold storage and supply chains	148
Leafy vegetables	Surface-enhanced Raman scattering (SERS) nanosensor	Organophosphorus pesticides	Sub-ppb to low ppb range	Rapid, label-free, highly sensitive pesticide residue detection on vegetable leaves	149
Vegetable extracts	Aptamer-functionalized electrodes	Multiple pesticides (*e.g.*, carbamates)	Low nM to pM levels	Highly selective and portable detection method with fast response	150
Leafy and fruit vegetables	Enzyme-inhibition electrochemical sensors	Organophosphates, carbamates	As low as 0.01 nM	Sensitive and rapid enzyme-based pesticide detection	151
Vegetable samples	Fluorescent nanosensors with nanomaterials	Pesticides and degradation products	Low ppb range	Effective detection of pesticide residue and chemical degradants	152
Various vegetables	Optical and electrochemical nanosensors integrated with AI analysis	Multiple pesticide classes	Not specified	Real-time, on-site monitoring with advanced data analytics	153

## 6. Conclusions

Nanotechnology-based sensors and nanomaterials are increasingly being used to improve food safety, quality, and sustainability by enabling rapid detection of contaminants and better monitoring of food products. This review highlights modern nanotechnologies for analyzing a wide range of food contaminants, including heavy metals, pesticides, pathogens, mycotoxins, and other harmful substances. These nanosensors use various nanomaterials that allow chemical and biological components to interact with target molecules and generate measurable signals. In food analysis, several inorganic nanoparticles, including titanium dioxide, gold, silver, zinc oxide, quantum dots, cerium nanoparticles, and magnetic nanoparticles, are widely used because of their excellent electrical conductivity, large surface area, magnetic properties, and adjustable physicochemical characteristics. Their large surface area also allows many biomolecules to be immobilized, increasing the number of binding sites available for target analyzes and improving detection efficiency. In addition to contaminant detection, nanotechnology supports food quality monitoring and shelf-life evaluation, making it valuable for early preventive action when food safety risks arise. Although these innovative nanosensors show strong potential for enhancing food quality and safety, further research is still needed to improve their practical application and broader implementation.

## 7. Challenges and future outlook

The main challenges to the use of nanosensors in food detection include complicated food matrix interference, high production costs, scalability, and regulatory requirements. Food matrices high in fats, proteins, and fibers typically result in non-specific interactions, lowering accuracy and repeatability in nanosensor detection systems. The complexity and expense of synthesizing carbon nanomaterials (CNMs) and other nanomaterials continue to be significant barriers to their practical widespread adoption. Furthermore, a lack of standardization in synthesis techniques causes sensor performance to vary between studies. Despite technological advancements, the absence of clear regulatory clearance paths remains a fundamental barrier for commercialization.^110,154,155^

With the development of nanosensors integrated into portable, user-friendly devices, including smartphone-based systems for on-site and real-time monitoring, the future is bright. The hybrid nanomaterials and synthesis methods using eco-friendly materials are being developed with an emphasis on scalability and sustainability. The integration of nanosensors with digital technologies, such as AI, IoT, and block chain, would empower food safety monitoring by real-time data analysis, predictive analytics, traceability, and rapid alert systems.^156^ Research efforts will be on multiplex biosensors that could detect multiple pathogens/toxins simultaneously, increasing detection efficiency. Interdisciplinary collaboration among nanotechnology, food microbiology, and material science would play an important role in overcoming current limitations and accelerating industry integration of this technology. These developments generally aim to guarantee food safety, reduce foodborne illnesses, and improve global public health.^157^

## Conflicts of interest

The authors declare no known conflicts of interest.

## Data Availability

Data sharing is not applicable to this article as no new data were generated or analyzed in this study.
